# Agmatoploidy and symploidy: a critical review

**DOI:** 10.1590/1678-4685-GMB-2016-0103

**Published:** 2016-10-27

**Authors:** Marcelo Guerra

**Affiliations:** Laboratório de Citogenética e Evolução Vegetal, Departamento de Botânica, Universidade Federal de Pernambuco, Recife, PE, Brazil.

**Keywords:** agmatoploidy, symploidy, holocentrics, holokinetic chromosomes, chromosome evolution

## Abstract

Agmatoploidy is a type of chromosome rearrangement that involves the fragmentation of an entire chromosome complement, generating a diploid with double its original chromosome number. Agmatoploidy and other related karyotype changes, such as symploidy (the opposite change, promoted by chromosome fusion), partial agmatoploidy, polyagmatoploidy, etc., are restricted to species with holokinetic chromosomes and are assumed to play an important role in their karyotype evolution. However, a critical review of the literature shows that examples of chromosome number doubling by agmatoploidy are rare and not clearly demonstrated, while partial agmatoploidy and partial symploidy seem to be the same as ascending and descending disploidy, respectively. It is therefore proposed here that these terms should be avoided or even abolished.

## Introduction

Chromosome number variations found in different species of a genus are usually due to disploidy (a chromosome rearrangement that results in a genomically similar karyotype that contains one or a few chromosomes more or less than that of the original) or to polyploidy (a cell division error that produces an entire genome duplication) (Guerra, 2008; Schubert and Lysak, 2011). In species with holokinetic chromosomes, however, two additional mechanisms are assumed to increase chromosome number variation: agmatoploidy (the fission of all of the chromosomes, resulting in a karyotype with a doubled chromosome number) and symploidy (concerted fusion, generating a karyotype with only half of the original chromosome number) (Luceño and Guerra, 1996). It is important to note that an agmatoploid is genomically identical to its diploid progenitor although the fragmented karyotype has distinct implications for the genetics and evolution of affected populations. It is not clear, however, what would make an entire chromosome complement suddenly split. Still more mysterious seems to be the opposite change: a two-by-two chromosome fusion of all of the non-homologous chromosomes (symploidy). The concept of agmatoploidy is revisited here in search for evidence that would support its occurrence in species with holokinetic chromosomes, and whether it should be considered a distinct mechanism of chromosome evolution.

## The original concepts

The term agmatoploidy (from Greek αγμα = fragment) was originally proposed by Malheiros-Gardé and Gardé (1950) based on their previous observations that the chromosome sizes of some "tetraploid *Luzula* were a little less than half of those of the diploid form and those of the octoploid species approximately half those of the tetraploids". As *Luzula* species have holokinetic chromosomes and X-ray-fragmented holokinetic chromosomes are regularly inherited (Castro *et al.*, 1949), the former authors proposed that the doubled chromosome complement was not the result of a polyploidization event but rather of fragmentation. Fragmentation had been previously reported in *Carex* species (Cyperaceae) by Heilborn (1924), based on careful chromosome length measurements. In *Carex*, chromosome numbers vary from *n* =6 to *n* = 66, including an almost continuous series of *n* =6 to *n* = 47 and more than 100 species presenting different cytotypes (Hipp *et al.*, 2009). Having in mind the taxonomic proximity of Cyperaceae and Juncaceae and the common occurrence of holokinetic chromosomes and inverted meiosis in *Carex* and *Luzula*, Malheiros-Gardé and Gardé (1950) proposed that this kind of fragmentation was a distinct evolutionary mechanism.

Nordenskiold (1951) observed that species of *Luzula* could have long (AL type), medium (BL type) or short (CL type) sized chromosomes, as in *Carex*. Species with 2*n* = 24BL that were closely related to species with 2*n* = 12AL appear to have arisen by agmatoploidy, whereas species with 2*n* = 24 AL were assumed to be polyploids. She also found karyotypes with "fragmentation" restricted to only few chromosomes, later denominated partial agmatoploidy (Love *et al.*, 1957). For example, in *Luzula spicata* the most common chromosome race had 2*n* = 24BL but there were a few samples with 2*n* = 14 (10AL + 4BL) and 2*n* = 12AL. Agmatoploid plants may also experience polyploidization, generating polyagmatoploids (Drábková, 2013).

The term symploidy (from Greek συν = together with), including complete simploidy and partial symploidy, was proposed by Luceño and Guerra (1996) as etymological correction for those cases in which chromosome number reductions associated with chromosome size increases were also referred to as agmatoploidy.

Similar variations in chromosome numbers and sizes or in DNA amount were later reported for several other species and genera, suggesting that agmatoploidy is an important mechanism of chromosome evolution in plants with holokinetic chromosomes (Hipp *et al.*, 2009; Bozek *et al.*, 2012; Bureš *et al.*, 2013; Drábková, 2013). It is important to observe that the term agmatoploidy, proposed by Malheiros-Gardé and Gardé (1950) in substitution to the expression "polyploidy by fragmentation", has been most commonly used in the sense of partial agmatoploidy instead of complete agmatoploidy. Actually, complete agmatoploidy generating a doubled chromosome number has been accepted only for some *Luzula* species. The occurrence of agmatoploidy in other genera of Juncaceae has been criticized because there is no evidence that they have holokinetic chromosomes (Schönswetter *et al.*, 2007; Drábková, 2013). In all other genera where agmatoploidy has been reported, including the large genera *Carex, Eleocharis, Schoenus,* and others, it was used to indicate partial agmatoploidy (Luceño and Castroviejo, 1991; Da Silva *et al.*, 2008; Kaur *et al.*, 2012). The same applies to the use of symploidy: complete symploidy has been reported for *Luzula* only, and all other reports of symploidy were considered as partial symploidy (see the same references above for agmatoploidy).

The interpretation of a karyotype change as agmatoploidy or symploidy depends on which karyotype/species is considered basal. For example, *Luzula spicata* subsp. *spicata* had 2*n* = 24BL whereas *L. spicata* subsp. *conglomerata* had 2*n* = 12AL, 2*n* = 16 (8AL + 8BL)*,* and 2*n* = 24BL (Nordenskiold, 1951). Assuming the most common cytotype, 2*n* = 24BL, as basal for *L. spicata*, the populations with 2*n* = 12AL and 2*n* = 16 (8AL + 8BL) would be considered symploids and partial symploids, respectively, whereas if the lowest number is the basal one than 2*n* =16 and 2*n* = 24BL should be, respectively, partial agmatoploid and complete agmatoploid.

## Agmatoploidy and nuclear DNA content

The assumption of evolution by agmatoploidy is restricted to cytotypes or species with doubled chromosome numbers and nearly conserved total chromosome length. Since total chromosome length or total chromosome volume correlates with nuclear DNA content (Barlow and Nevin, 1976, and references herein), agmatoploid species and cytotypes should have DNA contents quite similar to the original non-fragmented complements. This prediction was confirmed by early DNA content estimations based only on arbitrary values obtained from photometric studies in *Luzula* species (Halkka, 1964). However, Barlow and Nevin (1976) using microdensitometry of Feulgen stained nuclei and a standard species as control concluded that after the chromosome fragmentation there have been quantitative changes in the DNA content of members of the agmatoploid series, as later reported for other putative agmatoploid series (see *e.g.*, Zedek *et al.*, 2010).

When the concept of agmatoploidy was first established the tendency of polyploid species to reduce their genome sizes, and consequently chromosome lengths by rapid DNA losses (Leitch and Bennett, 2004), was still unknown. Currently, variation in DNA content among congeneric diploids and polyploids is widely known in plant taxa with monocentric or holokinetic chromosomes (reviewed by Bureš *et al.*, 2013). Therefore, conservation of DNA content and chromosome size is only expected in very recent rearrangements. Those reports of doubling chromosome numbers with DNA amount conservation must be investigate further, looking also for alternative explanations other than whole genome-wide chromosome fragmentation (Bačič *et al.*, 2007; Schönswetter *et al.*, 2007).

## Concerted chromosome fragmentation

In *Luzula*, the chromosome complement of some species or cytotypes look as they had been reduced to approximately half of their original size, maintaining the same symmetry of the non-fragmented parental karyotype (Malheiros-Gardé and Gardé, 1950; Nordenskiold, 1951). The mechanism promoting agmatoploidy must, therefore, operate at specific chromosome regions, sometimes denominated "fragile sites" (Roalson, 2008; Lipnerová *et al.*, 2013), splitting each chromosome into two halves of similar sizes. According to Malheiros-Gardé and Gardé (1950), "If the existence of these zones is confirmed, the hypothesis of agmatoploidy will be greatly reinforced". Detailed electron microscope and cytomolecular studies in *Luzula* (Heckmann *et al.*, 2011) and *Rhynchospora* (Marques *et al.*, 2015) species, however, did not reveal any differentiation along their chromosomes or kinetochores that could support this hypothesis. There is no known mechanism that could explain such concerted non-random chromosome fragmentation. Additionally, gamma irradiation of holokinetic chromosomes usually results in the formation of small chromosomes of different sizes (Tanaka and Tanaka, 1977; Sheikh *et al.*, 1995; Vanzela and Colaço, 2002; Jankowska *et al.*, 2015, suggestive of random fragmentation.

## Agmatoploidy and symploidy in the animal kindom

Another restriction to the existence of agmatoploidy is its exclusivity to plant species. Fragmentation of the whole chromosome complement had only been reported for very few animal species by the time this concept was proposed (see, *e.g*., Schrader and Hughes-Schrader, 1956). However, these data were associated with "multi-stranded" chromosomes (for a critique see White, 1973) and have never been confirmed or even mentioned in more recent reviews about holokinetic chromosomes written by animal cytologists (Mola and Papeschi, 2006; Mandrioli and Manicardi, 2012). Likewise, concerted fusion of all chromosomes has also been assumed for some taxa of Lepidoptera based only on the negative correlation between chromosome number and size in a taxon with holokinetic chromosomes (Saura *et al.*, 2013, and references herein). Actually, the term symploidy has never been used by animal cytologists.

Considering that holokinetic chromosomes have evolved independently nine times in animals and only four times in plants (Melters *et al.*, 2012), why would agmatoploidy occur in different plant clades but not in animals? The most reasonable explanation is that chromosome number duplications that were attributed to agmatoploidy in plants are in fact polyploids with reduced chromosome sizes. As polyploidy is rare or absent in most animal groups, reports of agmatoploidy in animals are also absent.

## How to distinguish partial agmatoploidy/symploidy from disploidy?

The term agmatoploidy has apparently been ascribed to several species simply because they had holokinetic chromosomes, without any clear criteria distinguishing it from polyploidy or disploidy. This is more evident for *Luzula* species, for which agmatoploidy was first described and has often been argumented as an important evolutionary mechanism (Bureš *et al.*, 2013; Drábková, 2013). Chromosome number doubling, genome size conservation, and pairing between normal and fragmented chromosomes during meiosis of hybrid plants bearing both chromosome complements are the minimal criteria required to presume agmatoploidy has occurred. Even in these cases, one should be able to discard the possibility that rapid and repeated ordinary fissions have occurred instead of concerted chromosome fragmentation.

The concepts of partial agmatoploidy and partial symploidy are indistinguishable from ascending and descending disploidy, respectively. All of them imply chromosome fusions or fissions without significant losses of chromatin. In both partial symploidy and descending disploidy, chromosome number reduction is promoted by breakage of two chromosomes followed by fusion of their broken ends, resulting in a larger chromosome, combining most or all genes of both chromosomes, and a small chromosome which is lost either because it is free of essential genes or lacks a stable centromere. In partial agmatoploidy or ascending disploidy, on the other hand, chromosome fission results from a single breakage followed by the addition of telomere repeats to the broken termini, usually mediated by telomerase or telomere capture (Schubert and Lysak, 2011). The acquisition of telomere repeats by the broken chromosome ends, necessary for the stabilization of the new chromosomes, was cytologically detected in *Luzula elegans* and other organisms a few cell generations after gamma irradiation (Jankowska *et al.*, 2015, and references herein). These types of chromosome rearrangements in both monocentric and holokinetic chromosomes may or may not be followed by significant changes in DNA content (Leitch and Bennett, 2004; Bureš *et al.*, 2013; Lipnerová *et al.*, 2013). Since no alternative mechanism has been proposed to explain chromosome fusion or fission in holokinetic chromosomes there is no reason to use a distinct terminology for holokinetics.

## Long disploid series may mimic complete agmatoploidy

Since fusions and fissions of holokinetic chromosomes do not affect their transmission to daughter cells, long disploid series and frequent intraspecific numerical variation are often found in genera with holokinetic chromosomes. The classical examples are *Carex*, which displays all chromosome numbers between *n* = 6 and *n* = 48 (Roalson, 2008), and *Luzula*, with an almost continuous series from *n* = 6 to *n* = 24 and other higher numbers (Drábková, 2013). Long disploid series are also found in several animal genera with holokinetic chromosomes. Among those taxa intensively investigated by genetic mapping and fine BAC-FISH mapping, such as *Caenorhabditis elegans*, *Bombyx mori*, and corresponding related species or genera, chromosome number variation has been demonstrated to be due to fission and fusion (dAlençon *et al.*, 2010). Gene-based comparative FISH mapping of the silkworm *B. mori* (2*n* = 56) and *Samia cynthia* (2*n* = 25-28), a moth species from a closely related family, showed that even a twofold variation in chromosome numbers most probably reflected repeated disploidy events (Yoshido *et al.*, 2011).

Large chromosome number variation, however, is not limited to genera having holokinetic chromosomes and is also found in genera of different families (Levin, 2002; Guerra, 2008). Likewise, extreme variation in chromosome number has been demonstrated by chromosome painting to be due to tandem fusions, as in the genera *Muntiacus* (deers), with 2*n* = 6/7 to 2*n* = 46 (Huang *et al.*, 2006), and *Akodon* (rodents)*,* with 2*n* = 10 to 2*n* = 46 (Ventura *et al.*, 2009).

## Conclusion

Although the concept of agmatoploidy has been widely accepted by plant cytogeneticists since it was first proposed over 65 years ago, there is still no compelling evidence that supports the occurrence of chromosome number duplication by the simultaneous fragmentation of all of the chromosomes. Complete agmatoploidy, complete symploidy, and polyagmatoploidy seem to be a misinterpretation of polyploidy. Partial symploidy and partial agmatoploidy are indistinguishable disploidy. All these terms should, therefore, be abolished or at least avoided until more convincing evidence has been presented.
